# Vaccine Images on Twitter: Analysis of What Images are Shared

**DOI:** 10.2196/jmir.8221

**Published:** 2018-04-03

**Authors:** Tao Chen, Mark Dredze

**Affiliations:** ^1^ Center for Language and Speech Processing Johns Hopkins University Baltimore, MD United States

**Keywords:** vaccine, visual communication, image tweet, Twitter, retweet prediction, social media

## Abstract

**Background:**

Visual imagery plays a key role in health communication; however, there is little understanding of what aspects of vaccine-related images make them effective communication aids. Twitter, a popular venue for discussions related to vaccination, provides numerous images that are shared with tweets.

**Objective:**

The objectives of this study were to understand how images are used in vaccine-related tweets and provide guidance with respect to the characteristics of vaccine-related images that correlate with the higher likelihood of being retweeted.

**Methods:**

We collected more than one million vaccine image messages from Twitter and characterized various properties of these images using automated image analytics. We fit a logistic regression model to predict whether or not a vaccine image tweet was retweeted, thus identifying characteristics that correlate with a higher likelihood of being shared. For comparison, we built similar models for the sharing of vaccine news on Facebook and for general image tweets.

**Results:**

Most vaccine-related images are duplicates (125,916/237,478; 53.02%) or taken from other sources, not necessarily created by the author of the tweet. Almost half of the images contain embedded text, and many include images of people and syringes. The visual content is highly correlated with a tweet’s textual topics. Vaccine image tweets are twice as likely to be shared as nonimage tweets. The sentiment of an image and the objects shown in the image were the predictive factors in determining whether an image was retweeted.

**Conclusions:**

We are the first to study vaccine images on Twitter. Our findings suggest future directions for the study and use of vaccine imagery and may inform communication strategies around vaccination. Furthermore, our study demonstrates an effective study methodology for image analysis.

## Introduction

### Background

Visual imagery plays an important role in communication in a range of domains. Their importance in health communication is widely acknowledged [1], and crafting effective health communication literature, materials, and campaigns involves creating visual content to extend the resonance of the message beyond the written word.

This is especially true in public health, where information awareness campaigns are one of the primary interventions available for changing public health behaviors; for example, the area of smoking cessation that has made extensive use of imagery in awareness campaigns. The *Tips from Former Smokers* campaign run by the Centers for Disease Control and Prevention used jarring imagery to encourage smoking cessation [2]. In addition, graphic warning labels on cigarette packages can dissuade people from smoking [3].

Images are especially important in communications related to vaccination, an area of public health with both proponents and opponents of the advocated behavior. Vaccine supporters and skeptics rely on scientific arguments and logic and emotional resonance to convince people of their perspective. Images are not only effective tools for eliciting emotional reactions but also for conveying statistics and data in support of a position. These can be combined in “infographics”—visuals that blend relevant imagery and statistics.

Both vaccine skeptics and supporters have a large presence on social media in general and Twitter in particular, and use these platforms to advocate for their positions [4,5]. Although several studies have looked at these communities, the topic of vaccine-related images has received little attention. The few studies [6,7] that have considered vaccine-related images have focused on *Pinterest*, an image-based social media platform. Although *Pinterest* is growing increasingly popular, it has less than half the total number of monthly active users as Twitter [8]. In addition, these studies leveraged qualitative analysis (ie, manual annotation), and thus their analysis is limited to a few facets for very small datasets. Moreover, they did not consider how image characteristics correlate with message engagement. See the Prior Work section in the following for a detailed review of the literature in this area.

In this paper, we examine a large corpus of vaccine-related image tweets. We are the first to study vaccine images on Twitter and pose two research questions:

- Research question 1: What are the common characteristics of vaccine-related images shared on Twitter?
- Research question 2: What properties of these images are correlated with higher engagement with other users?

To answer these questions, we pose and analyze various image properties via automated image analytics, which allows us to scale up our analyses to a large image collection. In addition, we fit a logistic regression model to model whether an image tweet was retweeted as a means to identify characteristics of images and tweets that are correlated with engagement. Our goals were to understand how images are used in vaccine-related tweets and to identify characteristics of vaccine images correlated with a higher likelihood of being retweeted.

### Prior Work

#### Images in General Public Health

Prior work on the effects of images in public health has focused on traditional media, such as brochures, advertisements, or magazines. Houts et al’s [1] comprehensive literature review summarized the following four functions of images in health communication: increasing patients’ *attention*, *comprehension*, *recall*, and *adherence* to health information. Images are most effective when they are closely linked to the written or spoken text and exhibit proper emotional stimuli. Effects can be more pronounced among low-literate people [9-11]. Houts et al [1] suggest the following seven guidelines for the effective use of images: (1) consider using images as visual aids; (2) prefer simple drawings or photographs to complex images; (3) simplify the accompanying text; (4) guide viewers toward an interpretation of the image; (5) be aware of the viewer’s culture; (6) take an active role in creating images; and (7) evaluate effects of images systematically.

There have been several analyses of images for specific use cases. Chang [12] studied the effects of images in advertising surrounding four diseases (tinea pedis, periodontal disease, H1N1, and peptic ulcers) and found that images can be effective in risk communication (ie, increasing audience’s perceptions of the severity of a disease) and educating the audience about prevention steps. In the same vein, pictorial warnings on cigarette packages have been shown to be a crucial factor for encouraging smokers to use cessation services and quit smoking and sustain their effects longer than text-only warnings [13-15].

Despite widespread acceptance that images are crucial in public health messaging, there is no visual theory that systematically guides the design and use of images, and characteristics of effective images in health communication remain unclear [16]. Furthermore, the community lacks standard tools to analyze health image content and estimate the effects on health behavior [16]. Our work aims to fill these gaps for vaccine images in social media. We identify the key features of vaccine images that correlate with image sharing through fitting a logistic regression model.

#### Images in Vaccination

To study the effectiveness of messages in a measles, mumps, and rubella (MMR) vaccine promotion (eg, textual information about the dangers of MMR diseases and images of sick children who have MMR diseases), Nyhan et al [17] conducted 2-wave Web-based survey experiments with 1759 parents who had children aged younger than 18 years. Unlike the positive effects of images in most public health studies, their experimental results showed that the image of a sick child had the opposite effect, that is, it increased parents’ beliefs in serious vaccine side effects. This result highlights the necessity of carefully testing vaccination messages before their use in a campaign.

The other three works studied vaccine images in social media, including *Facebook* [18] and *Pinterest* (an image-oriented platform) [7,6]. Broniatowski et al [18] analyzed news articles related to vaccine and vaccine-preventable illnesses during the Disneyland measles outbreak and measured the extent of several factors (eg, whether the article included a story and the article contained an image) that influence sharing on Facebook. They found that the presence of images in the article increases the likelihood of sharing, but they did not conduct an analysis of the images themselves.

Guidry et al [7] collected 800 vaccine-related pins (ie, posts from *Pinterest* that consist of an image and a caption) via keyword search and conducted a quantitative analysis to characterize content and user behaviors. In terms of the stance, the authors found that most pins (74.0%) portray vaccinations in a negative light, and antivaccine pins use more narrative than statistical information, whereas provaccine pins are just the opposite. A total of 81.5% of pins have an external reference (ie, contain an external URL), but only 0.3% refer to a government website and 3.7% refer to an official medical website (eg, hospital). They also examined the distribution of 5 Health Belief Model constructs in the dataset. For example, only 16.5% of pins perceived vaccinations to be highly effective, whereas 59.8% of pins showed that barriers to vaccination are high. For user behaviors, the most popular user engagement with a vaccine pin is repinning (a form of sharing), followed by “like” and comment.

Focusing on the images themselves, Milani [6] manually analyzed more than 1000 pins that clearly exhibited an antivaccination position. In all, 83.9% of the images were photos, 10.2% were charts and infographics, and the remaining 5.9% were drawings. In terms of the subject, syringes dominate (30.8% of pins), followed by children (19.8%), adults (14.6%), and the combination of children and syringes (11.7%). As such, syringes were the main semiotic sign of vaccination. They identified several stereotypes in the photos. Ethnically, all the physicians, paramedics, and 92.1% of babies appeared white. Emotionally, children often show neutral facial expressions when they are alone, smile when with family or in a group, and cry when taking a vaccination (with syringes) or portrayed as sick, whereas most adults are emotionless. Moreover, the author identified a common theme among the most repinned images, that is, rich in emotion but poor in information (ie, no textual information about vaccination). These posts use emotional appeal to try to persuade that vaccination is unnecessary and potentially harmful.

Although insightful, these previous studies have several limitations. Two of them [17,18] did not analyze the content of images, and the other two [7,6] conducted qualitative analysis of *Pinterest* images. As these works require manual labeling, their qualitative analysis is limited to a few facets for small datasets (less than a few thousand images). More importantly, none of them studied vaccine images on Twitter, a much more popular forum than *Pinterest*. In contrast, we rely on automated analytics to analyze millions of vaccine images on Twitter.

#### Vaccination on Twitter

Despite lack of prior work on vaccine-related images on Twitter, several studies have examined vaccine text tweets. A common thread is the study of attitudes and beliefs surrounding vaccination [4,5,19-21]. These studies typically leverage machine learning algorithms to automatically classify the sentiment (antivaccine, provaccine, or neutral) of vaccine tweets and then analyze the content based on this categorization. For instance, Dunn et al [19] found that users who were exposed to negative opinions about human papillomavirus (HPV) vaccine were more likely to subsequently post negative opinions, and Mitra et al [4] identified cohorts of users who persistently hold pro- or antivaccine attitudes via a longitudinal study.

Another line of research focuses on studying posts’ topics, dissemination patterns, community structures, and user behaviors [20,22-24]. For example, Surian et al [22] first characterized tweets about HPV vaccine using topic modeling and then examined the alignment of topics and user community structure. Radzikowski et al [24] collected vaccination tweets in the aftermath of the 2015 measles outbreak and analyzed key terms, the connection among such terms, communication patterns, and geographical patterns. Others have developed novel machine learning algorithms to discover tweets with specific traits, including identifying pseudoscientific claims about Zika vaccine [20] and inferring intentions (received or intend to receive) toward flu vaccine [23].

#### General Twitter Images

A relatively large number of tweets contain images (17.2%, according to a recent study [25]). Because image tweets are more likely to be shared than tweets without images, users are incentivized to add images to their messages. Unlike other photo-sharing websites, such as Flickr, Twitter images are not limited to photographs but include figures, graphics, screenshots, and other images meant to convey information or advertise the underlying content. For example, many people tweet images of articles that are too long to fit in a tweet or photos meant to accompany a linked website or news story. As a result, numerous studies have examined a range of aspects regarding images on Twitter (eg, [26]). Aspects have included characterizing images [27,28], automatically identifying sentiment of images [29,30], predicting image tweet popularity [31,32], detecting multimedia events [33,34], identifying fake images [35,36], mining trends from images [37,38], and understanding users [25,39].

## Methods

### Overview

We will characterize the types of images used in a vaccine-related message on Twitter. Our analysis relies on automated analytics for images and tweet content, and we identify factors that influence the likelihood that an image will be shared (retweeted). This section will describe dataset creation, content and image analytics, and our retweet prediction task.

### Dataset

#### Vaccine Twitter Data

**Figure 1:**
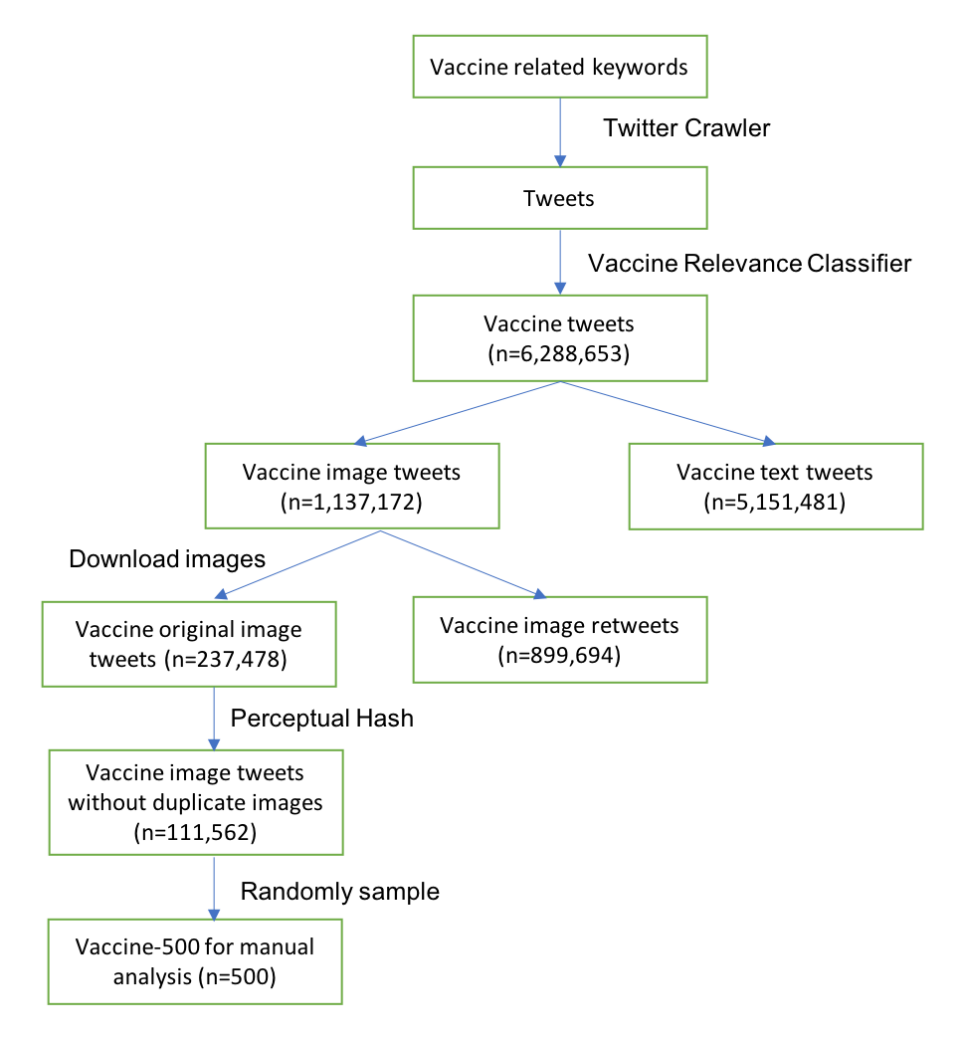
The flowchart of our vaccine Twitter data collection.

We constructed a large corpus of tweets relevant to vaccinations. Figure 1 shows the flowchart of the data collection. We first collected tweets that contained a term from a set of vaccine-related keywords and vaccine-preventable diseases (detailed in Textbox 1) using the Twitter streaming application programming interface (API) from November 11, 2014 to August 8, 2016. This includes all public tweets containing these keywords subject to a rate limit of roughly 1% of the total Twitter volume at that time.

We next applied a statistical classifier [40] to identify tweets relevant to vaccination, as opposed to irrelevant tweets containing vaccine keywords. This support vector machine (SVM) classifier was trained on 1899 manually annotated tweets (released at [41]) and achieved good performance (precision=0.96, recall=0.91, *F*_1_=0.93). After applying this classifier, we obtained a collection of 6,288,653 vaccine-related tweets (Table 1). A total of 18.08% (1,137,172/6,288,653) of the tweets contain an embedded image, a proportion similar to what has been previously reported (17.2%) for Twitter in general [25]. We obtained the number of times each original tweet was retweeted using the Twitter API [42] on December 11, 2016. We then downloaded all the images contained in the original tweets and referenced in the tweet metadata.

**The complete vaccine-related keywords and vaccine-preventable diseases used to query Twitter.**

vaccine, vaccines, mmr, tdap, flushot, hpv, polio, rotavirus, chickenpox, smallpox, hepatitis, hepa, hepb, dtap, meningitis, shingles, vaccinate, vaccinated, vaccine, vaccines, vacine, vacines, tetanus, diptheria, pertussis, whoopingcough, dtp, dtwp, chickenpox, measles, mumps, rubella, varicella, diphtheria, haemophilus, papillomavirus, meningococcal, pneumococcal, rabies, tuberculosis, typhoid, yellowfever, immunizations, immunization, imunization, immune, imune, cholera, globulin, encephalitis, lyme, zika

**Table 1:** The demographics of our vaccine tweet dataset.

Medium of tweet	Original tweet, n	Retweet, n	Total, n
Image tweet	237,478	899,694	1,137,172
Text tweet	3,162,184	1,989,297	5,151,481
Total	3,399,662	2,888,991	6,288,653

In addition, we randomly sampled 500 images (without duplicates, see below) from our large vaccine image tweet dataset (hereafter *vaccine–500*). This small dataset was primarily used to conduct manual analysis, which is complementary to the automatic analysis on the large dataset (detailed in the following section).

#### General Twitter Data

As a baseline of comparison for the retweet prediction task, we obtained a corpus of 200,000 general image tweets sampled from the Twitter 1% public feed between January 1 and December 12, 2016. Retweet counts were obtained on December 29, 2016, and 77.80% (155,600/200,000) of the image tweets had been retweeted at least once. We did not remove duplicates as they were rare in general image tweets.

#### News Data

The second baseline of comparison for the retweet prediction task will be images included in news articles related to vaccines. Following the procedure described in the study by Broniatowski et al [18], we collected 144,867 vaccine news articles from November 18, 2014 to November 15, 2016, from Google and Bing news using three keywords (vaccine, vaccination, and measles) associated with vaccination. We extracted article contents from HTML using *Goose* [43], including the main text and image of an article and filtered out articles without central images (eg, excluded logos, menu bar graphics, etc). This resulted in a set of 43,664 articles which had an image that was still accessible online. We then used the *Facebook* sharing API [44] to obtain each article’s share count, defined as the number of times the link has been shared on Facebook. We found that 51.51% (22,489/43,663) of the articles had been shared at least once. We did not remove duplicate images as they were rare.

#### Image Processing

##### Removing Duplicate Images

Many images appeared multiple times in our collection, either as exact copies of the same image file or different files with little change. We identified duplicate images using the Perceptual Hash [45], a popular algorithm for constructing fingerprints of images. Two photos will have similar hashes if they are nearly identical, for example, 2 images with identical content but different aspect ratios. To evaluate the performance of this algorithm, we manually checked 50 duplicate clusters (252 images in total) and found that it achieves 98.4% (248/252) accuracy. In our vaccine dataset, we found that 53.02% (125,916/237,478) of the images were near-duplicates. When we analyze a single tweet for an (duplicate) image that has multiple tweets, we select the tweet that is the most popular, as measured by retweet per follower (retweet count divided by follower count) [46]. This results in a set of 111,562 image tweets, of which 43.00% (47,972/111,562) have been retweeted.

##### User-Created Images

Users can tweet images that they create themselves, such as a picture taken by the user, but often times they distribute images that they obtain from other sources, such as an infographic or stock image. To differentiate between these images, we leveraged the Google Image Search query-by-image feature. We submitted each image in the *vaccine–500* set as a query and checked whether the image appeared on any other website.

### Image Analytics

#### Extracting Text From Images

**Figure 2:**
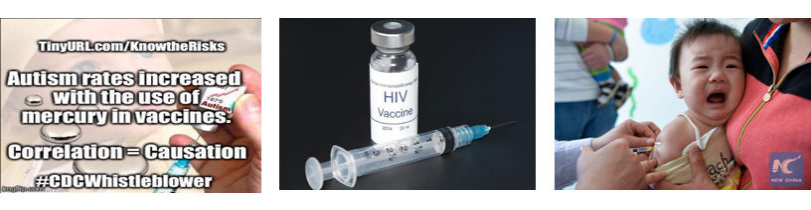
Three example vaccine images..

Many of the images contain text (see Figure 2 left for an example). Embedded text may be informative for interpreting the image [25]. We extract embedded text using *Tesseract* [47], an open source optical character recognition (OCR) toolkit. This tool is originally designed for printed text and thus works well for Twitter images similar to scanned text—detecting 89.5% of text-style images and 92.9% of screenshots that have text—and generally detects 68.4% of images with embedded text [25]. On the basis of the amount of text, we further categorize images containing embedded text into 3 groups: *primarily images* (no more than 10 words), *a mixture of an image and text* (between 10 and 30 words), and *primarily text* (more than 30 words).

#### Identifying Faces

Previous work found that many Twitter images contain pictures of people [27]. We identified and characterized human faces in images using *Face++* [48], an online face recognition tool. The tool identified faces and their estimated age, gender, and whether the person was smiling. *Face++* was reported to achieve 99.5% accuracy in face recognition [49], 83.0% accuracy in gender recognition, and have a mean absolute error at 11.0 for age estimation [50].

#### Object Recognition

We characterized the content of images using automated object recognition. We used *Clarifai* [51], a deep-learning powered commercial image recognition system. Clarifai provides textual tags to describe the content of an image and has a classification accuracy of 89.3% for the top 5 tags [52]. Consider the middle image in Figure 2 as an example. The top 5 tags from Clarifai are “syringe, injection, medicine, needle, and vaccination.”

#### Topic Analysis

We analyzed the content of tweets using Latent Dirichlet Allocation (LDA) [53], an unsupervised topic model that identifies major themes in a corpus through co-occurring words. This model has been widely used in the topic analysis for traditional text documents [53,54] as well as social media posts [22,55]. A key parameter of LDA is the number of topics, which could be determined on a held-out set.

In our work, we trained two separate LDA models to analyze post’s text and visual content. For the post’s text, we used the corpus of vaccine-related tweets (both text and image tweets) and trained the LDA model on the text of the post with 50 topics (textual topics, hereafter). For the visual content, we used the vaccine image corpus and trained LDA on the images’ tags provided by Clarifai with 40 topics (visual topics, hereafter). The number of textual or visual topics in both models was determined similarly by running an initial experiment with 20% of each dataset as a held-out set. LDA learns a topic distribution for each document and a word distribution for each topic. Following common practice [22,56], we used the topic with the largest probability as the document-level topic for a tweet or an image and manually assigned (by the first author of this paper) a label for each topic for clarity by looking at the top words in the word distribution. We then studied how the textual or visual topics correlate with the medium (image tweet or textual tweet), user engagement (retweeted or nonretweeted), and post’s sentiment.

#### Manual Analysis

In addition to the automatic image feature analysis, we manually examined the images from the *vaccine–500* corpus (conducted by the first author of this paper). This analysis is complementary to the automatic analysis and aims to further characterize the image content and shed light on the functions of images in vaccine messaging.

### Retweet Prediction

What makes a vaccine image tweet compelling or engaging? To answer this question, we consider a proxy task: What characteristics of the text and embedded image make it more likely to be shared? We frame this as a retweet prediction task, where we use binary classification to determine whether a tweet was retweeted or not (yes or no). To the best of our knowledge, we are the first to study retweet prediction for vaccine image tweets. Most prior studies concentrate on general text tweets [46,57-59], and only two have specifically considered general image tweets [31,32].

Our study uses a logistic regression, a generalized linear model, to estimate the probability of a binary response (in our case, retweeted or not). Logistic regression was used in previous work for the retweet prediction [57,58,46] and has a key advantage of interpreting the predictive power of features via computing odds ratio. To build a retweet classifier, the previous work exploited various features from tweet’s textual content (eg, words, topics, and sentiment), contextual metadata (eg, posting time, the presence of URL), author’s profile (eg, the number of followers and friends), and images (eg, color histograms, GIST descriptors, detected visual objects). In our study, we extracted a wide spectrum of features inspired by the previous work and additionally proposed several vaccine-specific features (described below).

To evaluate the model performance, we split our vaccine image tweet dataset (111,562 image tweets after removing duplicate images) into training and test set. To model the timeliness of tweets, we keep the most recent 20% tweets as test set and the rest 80% older tweets as training set. We report precision (true positives divided by true and false positives), recall (true positives divided by true positives and false negatives), and the *F*_1_ score (harmonic mean of precision and recall). Following the same setting, we also build predictive models for the vaccine news and general image tweet dataset by using the same set of features (excluding vaccine-specific features).

#### Features Used for Predictive Model

##### Author Popularity Features

One of the most important predictors of whether a tweet will be retweeted is the popularity of the tweet’s author [31]. To control for author popularity, we added two features to the model: (1) whether or not the user account is verified (Twitter’s account verification establishes account authenticity for public figures or those at risk of impersonation) and (2) the follower count (log normalized). For vaccine news, we measured the author popularity by the Facebook share count of its URL domain (not the link to the story itself).

##### Metadata Features

We extracted three features from a tweet’s metadata: (1) the number of included hashtags, (2) user mentions, and (3) images. We added features indicating the presence of a URL in the tweet, whether the link was to a government (*.gov*) or nongovernment page, and whether the page was no longer available. We similarly classified vaccine news as government or nongovernment page based on the URL.

#### Text Features

We considered three types of text features.

- Topic: We added features based on the inferred LDA topic model, where each feature is the probability of a topic in the document. Considering that the tweet’s text is short, we followed previous work [25] to obtain an enriched textual representation for a tweet by combining (1) the tweet’s text, (2) the text from webpages that were linked in a tweet, and (3) the embedded text from the images. We then trained the LDA topic model based on the enriched text. For vaccine news, we combined the OCR text with its article content. We then trained separate topic models for the three datasets.
- Vaccine names: We identified whether the tweet contained one of 25 vaccine names (eg, MMR, HIV) that could suggest the vaccination topic of the tweet. This feature is applied to vaccine image tweets and news but not for general image tweets.
- Sentiment: Previous work found that sentiment is a predictor of retweets [58]. To identify the sentiment of vaccine tweets, we built two SVM classifiers. We first trained a classifier with 447 labeled tweets to identify sentimental tweets from neutral tweets, and then we trained another SVM classifier with 153 labeled tweets to identify sentimental tweets as provaccine or antivaccine. These two classifiers obtain an *F*_1_ score of 0.80 and 0.39 on the test set, respectively. To facilitate further research, we have released the labeled dataset at [41]. We included this sentiment label (neutral, provaccine, or antivaccine) as a feature. Due to the lack of proper tools, we did not extract this feature for general image tweets and vaccine news.

#### Image Features

We extracted features from the image that capture high-level semantics and low-level vision properties.

- Visual topics: We used the topics learned by fitting LDA to object recognition tags provided by Clarifai. We added topics in the same manner described previously for text topic features. Considering the images in general tweets differ significantly from vaccine images, we restrict these features only to vaccine image tweets and news.
- Face recognition: We extracted four face-related features: (1) number of faces, (2) gender (does the image have a male or female face), (3) age group (does the image have a face with an age falls in 0-2, 3-14, 15-24, 25-64, or over 65 years), and (4) smiling face.
- Image type: We identified images as *pure image*, *primarily image*, *a mixture of image and text*, and *primarily text* as defined previously.
- Visual sentiment: Low-level image features have been shown to be a simple but an effective way to capture emotions, sentiment, or effect of an image [28,60]. We extracted five sets of color-based features to capture visual sentiment: (1) saturation: the mean and standard deviation of saturations; (2) brightness: the mean and standard deviation of brightness; (3) hue: the mean and angular dispersion, with and without saturation weighted; (4) dominant color: the most prevalent of 11 basic colors (ie, black, blue, brown, green, gray, orange, pink, purple, red, white, and yellow) [61]; and (5) affectiveness: one set of three affective scores to measure the pleasure, arousal, and dominance [62].

## Results

### Analysis of Vaccine Image Tweets

#### Image Tweet Corpus Analysis

Although Twitter allows users to attach up to 4 images per tweet, most vaccine image tweets (1,089,411/1,137,172; 95.80%) have a single image. These images make the vaccine tweets more likely to be shared (72,906/237,478; 30.70% of image tweets were retweeted) than their text-only counterparts (483,815/3,162,184; 15.30%). While the text-only tweet retweeting rates are similar in the general tweet dataset (13.61%; 10,379/76,273 retweeted), a huge difference exists in image tweets, that is, general image tweets are 2.5 times (155,600/200,000; 77.80%) more likely to be shared than vaccine image tweets. This highlights the need to understand how images are used to discuss vaccines on Twitter and identify strategies that lead to effective images.

Most of the images were not user created but were instead prepared infographics, stock photos, or other imagery. In the *vaccine–500* corpus, 88.4% (442/500) of the images were found on other websites, suggesting that they were not user generated. Users tweeting about vaccines are sharing existing images, which may explain why so many images are reused by other users. In addition, a large number of vaccine images contain text; 39.90% (44,513/111,562) of vaccine images contained at least one embedded textual word. Of which, 42.90% (19,096/44,513) were *primarily images* (no more than 10 words), 30.60% (13,620/44,513) were *a mixture of an image and text* (between 10 and 30 words), and 26.50% (11,797/44,513) were *primarily text* (more than 30 words).

We also found that one-fourth (28,560/111,562; 25.60%) of vaccine images contain faces. The majority of these had a single face, and most of the remaining images (4798/28,560; 16.80%) had 2 faces. The large presence of faces agrees with the objects and concepts discovered by object recognition; four of the five most frequent Clarifai tags (“people, business, adult, woman, and man”) are explicitly about people.

#### Topic Analysis

**Figure 3:**
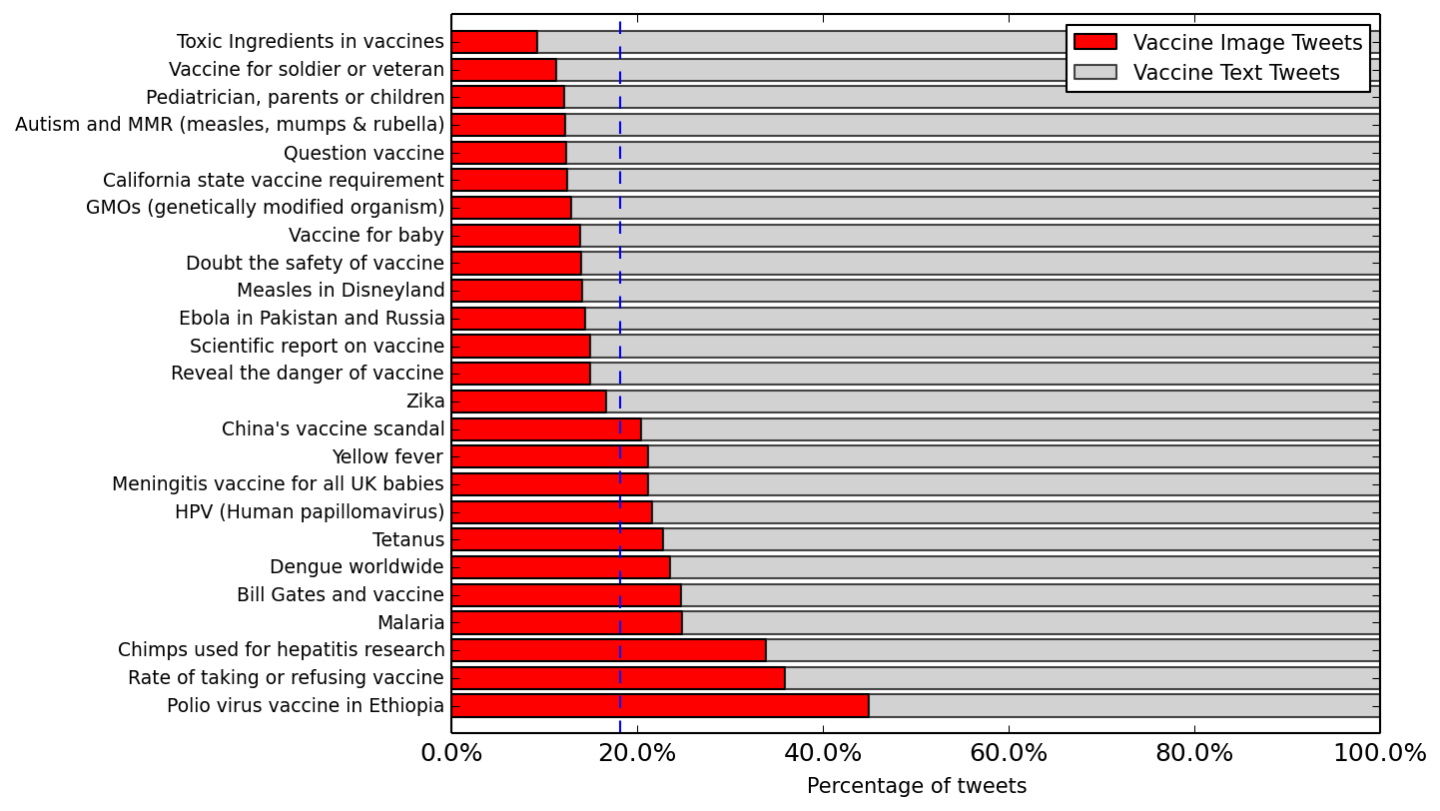
The distribution of vaccine image tweets and text tweets in the selected textual topics. On average, 18.08% (1,137,172/6,288,653) of vaccine tweets are image tweets (indicated by the vertical line).

Our topic model analysis (without retweets) showed that images were more likely to be associated with some textual topics. Figure 3 lists the manually assigned label for 25 of the topics that were found to be semantically coherent and had the highest deviation of the proportion of tweets containing an image from the mean. The vertical line indicates the mean across the corpus. The topics “poliovirus vaccine in Ethiopia,” “chimps used in hepatitis research,” and “the rate of taking or refusing vaccine” had the highest proportion of image tweets, whereas tweets about “toxic ingredients in vaccines” and “vaccine for soldier or veteran” had the lowest.

**Figure 4:**
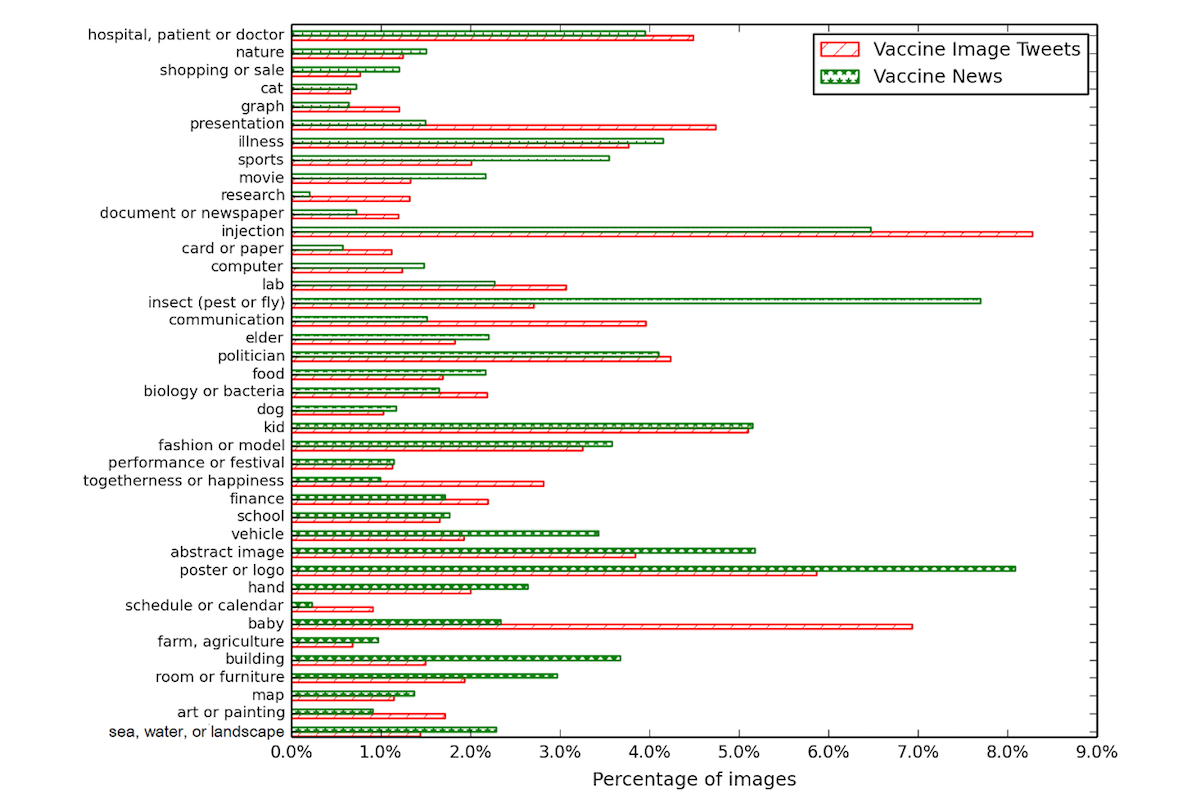
The topic distribution of vaccine image’s visual tags.

**Table 2:** The most dominant visual topic and its percentage in each textual topic.

Textual topic	Dominant visual topic	n (%)
Dengue worldwide	Insect (pest or fly)	334 (24.29)
Vaccine price	Poster or logo	231 (17.88)
Poliovirus vaccine in Ethiopia	Baby	3930 (91.43)
HPV^a^	Injection	376 (13.60)
H1N1, swine, or bird flu	Injection	275 (21.43)
Malaria	Insect (pest or fly)	342 (21.30)
Vaccines and fertility	Female	114 (71.5)
Debates of Republican candidates on vaccines and autism	Politician	852 (20.97)
Chimps used for hepatitis research	Nature	1021 (56.07)
Rabies	Dog	1585 (36.91)
Zika	Insect (pest or fly)	534 (23.56)
Meningitis vaccine for all UK babies	Baby	373 (15.84)
Autism and MMR^b^	Communication	410 (16.18)
Scientific report on vaccine	Biology or Bacteria	299 (14.06)
Genetically modified organism	Food	133 (7.53)
Ebola in Africa	Hospital, patient, or doctor	473 (17.81)
Anticancer vaccines	Abstract image (cell or cartoon)	182 (12.17)
HIV	Poster or logo	147 (10.55)
Vaccine for soldier or veteran	Vehicle	37 (6.9)
Promote vaccine	Baby	255 (10.36)

^a^HPV: human papillomavirus.

^b^MMR: measles, mumps, and rubella.

Turning to the topic model analysis of the Clarifai visual tags, the most common topics we found were injection (Figure 2, left) and baby (Figure 2, right). For comparison, we additionally applied the same procedures to the vaccine news images. Tweets and news exhibit differences in using images (see Figure 4). Compared with the visual tags for Twitter, poster or logo and insect are the most used images in news, which takes up 8.08% (3258/43,664) and 7.70% (3362/43,664) of news images, respectively. We then measured the correlation between the text topic and visual topic in the same tweet. Table 2 lists the most common visual topic for each of the 20 textual topics, with the proportion of images in a tweet and the text topic containing the visual topic. We observe strong semantic connections: posts about Dengue show images of insects, posts about polio show babies, and posts about HPV show injections.

**Figure 5:**
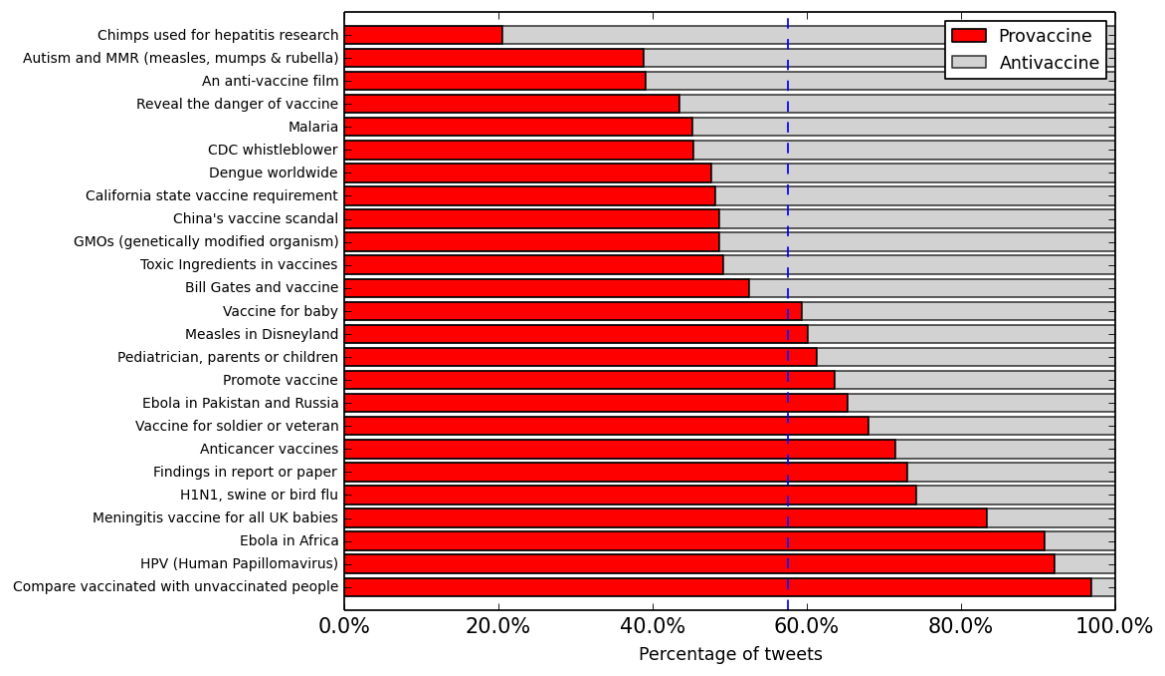
The sentiment distribution of vaccine image tweets' textual topics. On average, 57.58% (71,417/124,029) of sentimental vaccine image tweets are provaccine (indicated by the vertical line). CDC: Centers for Disease Control and Prevention.

**Figure 6:**
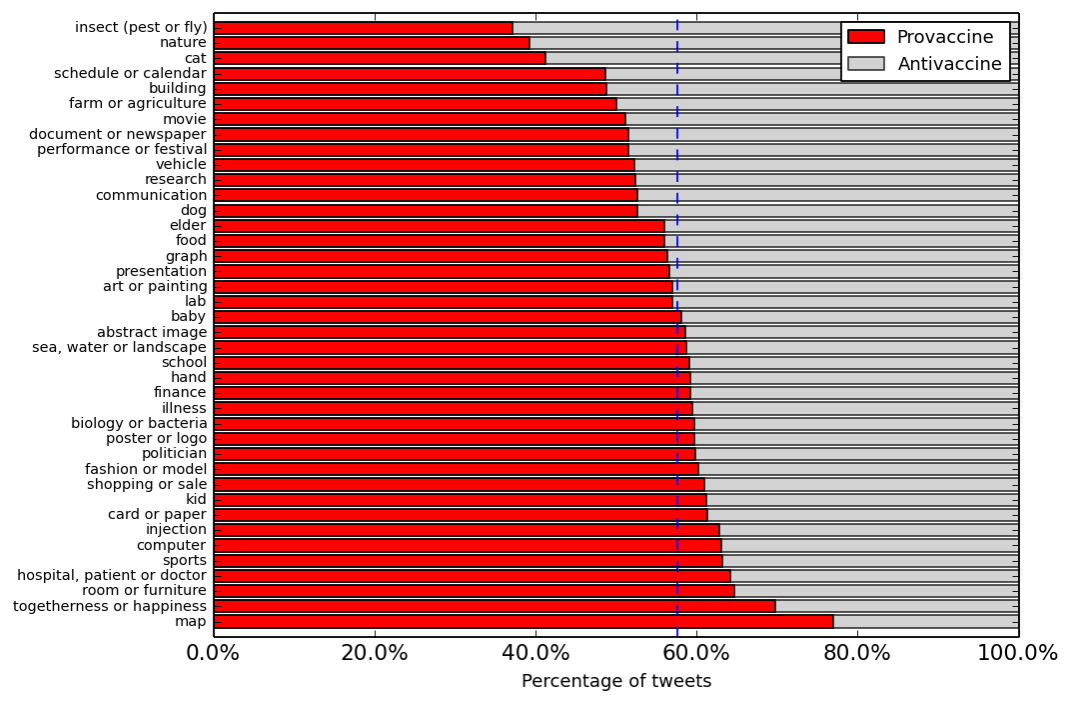
The sentiment distribution of vaccine image tweets' visual topics. On average, 57.58% (71,417/124,029) of sentimental vaccine image tweets are provaccine (indicated by the vertical line).

Finally, we studied the correlation between the topic and sentiment. We plot the sentimental distribution within each textual and visual topic in Figures 5 and 6, respectively. From Figure 5, we see that many textual topics show skewed sentiment distribution. People primarily express provaccine attributes when they discuss the topic of “comparing vaccinated with unvaccinated people,” “HPV,” “Ebola in Africa,” and “Meningitis vaccine for all UK babies,” while expressing antivaccine sentiment in the discussion of “Chimps used for hepatitis research,” “an antivaccine film,” and “Autism and MMR.” On the contrary, visual topics exhibit more balanced sentimental distribution, as most topics are close to the averaged sentimental distribution (Figure 6).

#### Manual Analysis

We found that most images (without the associated tweet’s text) are indicative of the topic of vaccine or health (eg, Figure 2, middle). These images function as a *semiotic sign*, making the tweet differentiable from the huge amount of nonvaccine tweets on Twitter. For the people in the images, we noticed many of them were taking an injection, which further implies the tweet is discussing the vaccination for that specific group of people (Figure 2, right). We then turn to the images that contain text. Such images are an indispensable component of the tweets, either displaying long text as an image to overcome Twitter’s text length restriction (up to 140 characters) or render key information of the tweet visually (eg, charts and figures). Aside from these, we identified the third use case of vaccine images that exhibit strong emotion (Figure 2, right), which is used to enforce the sentiment of the post. In summary, we identified three key functions of vaccine images: (1) expressing the topic visually, (2) supplementing information in the tweet, and (3) eliciting emotional responses.

**Table 3:** Experimental results of sharing prediction. Large score denotes better performance.

Dataset	Precision	Recall	*F*_1_
Vaccine image tweets	0.713	0.632	0.670
Vaccine news	0.750	0.596	0.683
General image tweets	0.856	0.947	0.899

### Retweet Prediction

The logistic regression classifier obtained an *F*_1_ score of 0.670 (precision=0.713, recall=0.632) when predicting if a vaccine tweet would be retweeted. For comparison, we conducted similar experiments for vaccine news and general image tweets. Table 3 shows that the predictive model achieves much better performance on general image tweets (*F*_1_=0.899) than vaccine image tweets (*F*_1_=0.670) and news (*F*_1_=0.683). This implies retweet prediction in the focused vaccine domain is more challenging than the general domain.

We computed the odds ratio of the coefficients to determine the strength of a feature in predicting retweets. We compared the features among the three datasets when applicable. For both tweet datasets, the metadata features positively predict retweets, and information about the author (eg, their popularity) matters most (Table 4).

Table 5 shows the statistically significant text features for vaccine tweets and news. Mentioning some vaccines by name increases retweeting (HPV, pertussis, polio, and smallpox), whereas others decrease retweeting (Zika, flu, anthrax, and meningococcal). For news, six vaccine names exhibit strong indication for either sharing (meningococcal, HPV, and typhoid fever) or not sharing (shingles, Zika, and adenovirus). Expressing emotion (either pro- or antivaccine) in the text makes the vaccine tweet more retweetable. Although sharing URLs does not predict retweets, nongovernment and deleted URLs more negatively indicate retweets than government URLs. For news, the articles from government site are significantly more shared than other news. Finally, the textual topics play a key role in retweeting. Tweets that discussed topics such as “Scientific report on vaccine,” “Yellow fever,” “Trial,” and “Vaccine for soldier or veteran” attract more retweeting, whereas other topics such as “Dengue worldwide,” “Autism and MMR,” “Poliovirus vaccine in Ethiopia,” “Ebola in Pakistan and Russia,” and “Chimps used for hepatitis research” are negative indicators for retweeting.

**Figure 7:**
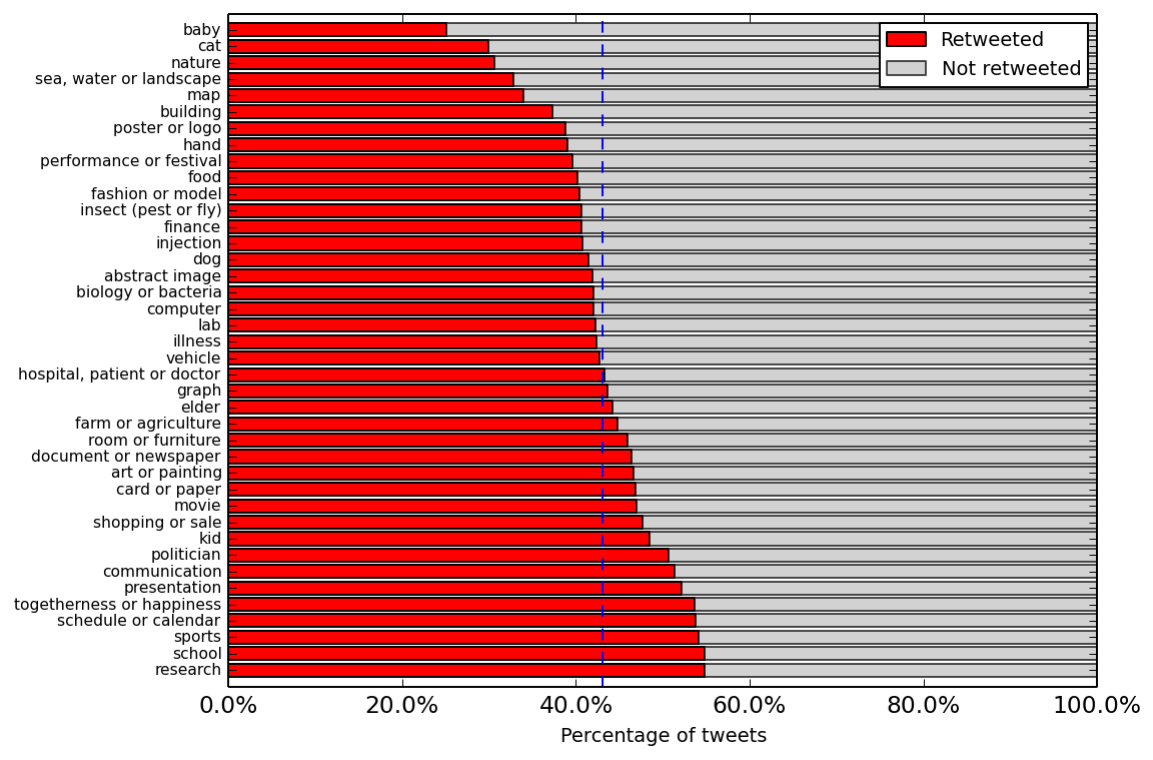
After image deduplication, the proportion of retweeted and nonretweeted vaccine image tweets in each visual topic. On average, 43.00% (47,972/111,562) of vaccine image tweets have been retweeted (indicated by the vertical line).

We finally turn to the features based on images (Table 5) and focus on the comparisons of vaccine tweets and news. In general, the high-level visual features are very predictive for both tweets and news. Figure 7 shows the proportion of retweeted and nonretweeted vaccine image tweets in each visual topic. Looking at the specific topics, the predictive topics are rather different on Twitter and Facebook, and some topics have contrary predictive power. For example, images of communication and sports increase retweeting but decrease news sharing on Facebook. Images with faces are not predictive in general, but the presence of a smiling face is a positive indicator for retweeting. Embedding some text in the image always increases retweeting regardless of the amount of text but does not have a significant impact news sharing. In general, visual sentiment features are predictive for both tweet and news, but the specific features and their impact often differ in the two datasets. Such feature discrepancies suggest that vaccine visual communication may differ between platforms (Twitter and Facebook) and mediums (tweet and news).

**Table 4:** The odds ratios of Twitter metadata features that are statistically significant.

Feature	Vaccine image tweets		General image tweets	
	OR^a^ (95% CI)	*P* value	OR (95% CI)	*P* value
Follower count	2.55 (0.92 to 0.96)	<.001	4.78 (1.54 to 1.58)	<.001
Verified user	3.55 (1.19 to 1.34)	<.001	2.98 (0.97 to 1.20)	<.001
Mention count	1.33 (0.26 to 0.31)	<.001	1.39 (0.30 to 0.35)	<.001
Image count	1.15 (0.09 to 0.18)	.01	1.69 (0.50 to 0.56)	<.001
Hashtag count	1.24 (0.20 to 0.23)	<.001	1.05 (0.03 to 0.06)	<.001

^a^OR: odds ratio.

**Table 5:** The odds ratios of textual and image features that are statistically significant.

Feature	Vaccine image tweets			General image tweets		
	Feature	OR^a^ (95% CI)	*P* value	Feature	OR (95% CI)	*P* value
Vaccine names	HPV^b^	1.24 (0.05 to 0.29)	<.001	HPV	1.20 (0.00 to 0.36)	.05
	Zika	0.76 (–0.43 to –0.11)	<.001	Zika	0.63 (–0.59 to –0.34)	<.001
	Meningococcal	0.56 (–0.72 to –0.45)	<.001	Meningococcal	1.28 (0.11 to 0.39)	<.001
	Pertussis	1.34 (0.14 to 0.45)	<.001	Shingles	0.71 (–0.63 to –0.06)	.02
	Polio	1.27 (0.15 to 0.33)	<.001	Adenovirus	0.55 (1.08 to –0.12)	.01
	Smallpox	1.65 (0.24 to 0.77)	<.001	Typhoid fever	1.70 (0.06 to 0.99)	.03
	Flu	0.85 (–0.23 to –0.09)	<.001			
	Anthrax	0.72 (–1.02 to 0.37)	.008			
Textual sentiment	Neutral	0.87 (–0.29 to –0.21)	<.001	N/A^c^		
URL reliability	Government URL	0.92 (-0.23 to 0.07)	<.001	Government URL	1.97 (0.71 to 1.79)	<.001
	Nongovernment URL	0.76 (-0.32 to -0.24)	<.001			
	Deleted URL	0.48 (-0.81 to -0.68)	<.001			
Textual topics	Autism and MMR^d^	0.76 (-0.73 to -0.41)	<.001	Omitted as a separate topic model was trained for news and the comparisons may not be meaningful.		
	Dengue worldwide	0.70 (–0.84 to –0.45)	<.001			
	Poliovirus vaccine in Ethiopia	0.12 (–2.61 to –2.14)	<.001			
	Vaccine for soldier or veteran	1.55 (0.12 to 0.42)	<.001			
	Scientific report on vaccine	1.60 (0.01 to 0.35)	.002			
	Ebola in Pakistan and Russia	0.73 (–0.80 to 0.41)	<.001			
	Chimps used for hepatitis research	0.42 (–1.39 to –0.95)	<.001			
	Yellow fever	1.41 (–0.12 to 0.23)	<.001			
	Trial	1.79 (0.06 to 0.52)	<.001			
Visual topics	Art or painting	1.21 (0.08 to 0.36)	<.001	Sea, water, or landscape	1.21 (0.03 to –0.48)	.03
	Communication	1.28 (0.14 to 0.40)	<.001	Communication	0.57 (–0.78 to –0.25)	<.001
	Sports	1.45 (0.25 to 0.53)	<.001	Sports	0.72 (–0.48 to –0.07)	.01
	School	1.35 (0.18 to 0.47)	<.001	Building	0.76 (0.40 to –0.02)	.03
	Schedule or calendar	1.38 (0.18 to 0.51)	<.001	Fashion, model, or female	1.31 (0.1 to –0.55)	<.001
	Togetherness or happiness	1.28 (0.13 to 0.41)	<.001	Biology or bacteria	1.60 (0.27 to 0.79)	<.001
	Performance or festival	0.76 (–0.47 to –0.16)	.03	Food	1.51 (0.24 to 0.70)	<.001
	Politician	1.38 (0.22 to 0.47)	<.001	Elder	1.29 (0.08 to 0.56)	.01
	Hand	0.77 (–0.40 to –0.08)	.003			
	Research	1.37 (0.20 to 0.48)	<.001			
	Presentation	1.26 (0.14 to 0.38)	<.001			
	Graph	1.46 (0.24 to 0.57)	<.001			
	Cat	0.34 (–1.20 to –0.92)	<.001			
Face	Smile	1.27 (0.16 to 0.32)	<.001			
	Primary image	1.24 (0.17 to 0.27)	<.001			
	Mixture	1.16 (0.09 to 0.20)	<.001			
	Primary text	1.34 (0.22 to 0.37)	<.001			
Visual sentiment	Blue	0.84 (–0.83 to –0.27)	<.001	Blue	1.87 (0.20 to 1.21)	.006
	White	0.84 (–0.79 to –0.30)	<.001	Red	0.35 (–1.52 to –0.45)	<.001
	SD of Brightness	2.16 (0.49 to 1.05)	<.001	SD of Brightness	0.52 (–1.10 to –0.20)	.005
	Weighted mean of hue	5.65 (1.04 to 2.42)	<.001	Weighted mean of hue	0.08 (–3.70 to –1.41)	<.001
	Arousal	0.73 (–1.33 to –0.40)	<.001	Arousal	1.38 (0.15 to 1.65)	.02
				SD of Saturation	1.79 (0.17 to 1.00)	.006

^a^OR: odds ratio.

^b^HPV: human papillomavirus.

^c^N/A: not applicable.

^d^MMR: measles, mumps, and rubella.

## Discussion

### Overview

Images have been largely used in vaccine tweets (1,137,172/6,288,653; 18.08% of vaccine tweets contain an image) but were neglected by the prior work. We are the first to study vaccine images on Twitter and particularly answer two research questions: (1) what are their characteristics? and (2) what are the kinds of traits that make them engaging? We summarize the key findings and their implications and highlight some future works in the following paragraphs.

### Principal Findings

As with the previous work on general image tweets, an image makes a vaccine tweet more likely to be shared (72,906/237,478; 30.70% of image tweets were retweeted) than their text-only counterparts (483,815/3,162,184; 15.30%). This is a possible motivation for users to attach images to tweets [26,63]. The large number of vaccine images from Twitter that are duplicates (125,916/237,478; 53.02%) or found on other websites (442/500, 88.4%) suggests that most vaccine images are not user created (eg, a photo taken by a user); instead, they are selected from other sources by the user to help promote their vaccination message. Also, the much higher proportion of vaccine image tweets with external URLs (653,874/1,137,172; 57.50%) compared with general image tweets (22.7% [25]) suggests that images play an important role in vaccine-related messaging. This makes Twitter an especially attractive platform for assessing the effectiveness of vaccine visual communication.

Furthermore, many of the vaccine images contain their own information beyond a visual supporting of the message in the tweet’s text. Nearly 40% of images have embedded text, and embedded text is informative to interpret the overall message of the tweet. These images include screenshots, infographics, charts, and figures, which is consistent with vaccine images on *Pinterest* [6,7]. Focusing on the visual content, the two most recurring objects in vaccine images are syringes and people, which is consistent with the findings on *Pinterest* [6]. The visual content is also highly correlated with a tweet’s textual topics. As such, the purpose of attaching an image to a tweet is to make it more attractive [27] and convey the topics of the tweet.

Vaccine tweets with images were twice as likely to be shared as nonimage tweets, which follows the trend of general tweets [26]. Our logistic regression identified the author as one of the most important factors for determining whether an image tweet would be shared, the same trend as in general tweets [46,57], and consistent with the findings in the study by Broniatowski et al [18]. Sentiment features, extracted from text and image, are also predictive of sharing behavior. Positive or negative sentiment vaccine image tweets are more likely to be retweeted than neutral tweets, which also matches the behavior of *Pinterest* [6]. One-fourth of vaccine images contain faces. Although previous work found that images with faces have a higher user engagement [64], we found that vaccine image tweets containing a face were equally likely to be retweeted as those without a face (25.5% with vs 25.7% without).

Comparison between retweet prediction for general image tweets and vaccine news shows that retweet prediction for vaccine image tweet is a much more difficult task. We found differing behaviors of features between vaccine tweets and vaccine news. For instance, a smiling face increased sharing for vaccine tweets, but not news, whereas pictures of landscape and nature contribute positively for news sharing but negatively for tweets. This suggests that different communication patterns exist in the two domains (tweet and news), or there could be a difference in how people decide to share content on the two social media platforms (Twitter and Facebook).

### Implications

Our research has implications for public health researchers and practitioners.

We demonstrated that images are widely used in Twitter vaccine messages and characterized these images using several types of analyses. This should aid in understanding the information content of millions of vaccine tweets.

In addition, vaccine-related communication strategies could benefit from our analyses. Images boost the reach of a vaccine message. Our retweet predictive model could be used as a tool to assess the effectiveness of designed visual vaccine messages. From that model, we also identify a few key factors that correlate the retweeting of vaccine tweets. Although we have not established a causal relation, these factors could still guide message design.

Finally, our study demonstrates an effective methodology for image analysis studies. We found that Twitter is a productive platform for studying visual communication issues surrounding vaccines. This is an important finding because Twitter makes it relatively easy to collect large quantities of image data via the public Twitter API, compared with the lack of *Pinterest* APIs for creating large, unbiased datasets [65]. Unlike prior work that relied on human analysis of images, we used fully automated analytics to conduct a comprehensive analysis over a large dataset. Such techniques can be applied to analyze vaccine images from other sources and health-related images in general. To enable future studies, we have released the labeled datasets that were used to build our vaccine relevance and sentiment classifiers [41].

### Future Directions

We see several avenues of future work. Although our study of Twitter adds to other work that has studied Pinterest, several large social media platforms, in which images are prevalent, have not been examined for vaccine content. These include Instagram and Facebook. Because effective messaging strategies need to be tailored for each platform, evidence of vaccine image effectiveness on these platforms would be welcome.

In addition, we are interested in understanding images beyond the analytics presented in this paper. For example, what images are most effective for different campaigns? How do images tie into existing narratives around vaccination? How are target populations of vaccination campaigns reflected in images? Finally, these questions can be applied broadly to public health awareness campaigns. We plan to extend our methodology to consider these questions in future work.

## Abbreviations

API: application programming interface
HPV: human papillomavirus
LDA: Latent Dirichlet Allocation
MMR: measles, mumps, and rubella
OCR: optical character recognition
SVM: Support Vector Machine
